# Should Public Health and Policy Communities Interact With the Food Industry? It Depends on Context Comment on "Towards Preventing and Managing Conflict of Interest in Nutrition Policy? An Analysis of Submissions to a Consultation on a Draft WHO Tool"

**DOI:** 10.34172/ijhpm.2020.176

**Published:** 2020-09-20

**Authors:** Katherine Cullerton, Jean Adams, Martin White

**Affiliations:** ^1^School of Public Health, University of Queensland, Herston, QLD, Australia.; ^2^MRC Epidemiology Unit, University of Cambridge School of Clinical Medicine, Cambridge, UK.

**Keywords:** Nutrition, Health Governance, Conflict of Interest, Food Industry, Policy-Making

## Abstract

The issue of public health and policy communities engaging with food sector companies has long caused tension and debate. Ralston and colleagues’ article ‘Towards Preventing and Managing Conflict of Interest in Nutrition Policy? An Analysis of Submissions to a Consultation on a Draft WHO Tool’ further examines this issue. They found widespread food industry opposition, not just to the details of the World Health Organization (WHO) tool, but to the very idea of it. In this commentary we reflect on this finding and the arguments for and against interacting with the food industry during different stages of the policy process. While involving the food industry in certain aspects of the policy process without favouring their business goals may seem like an intractable problem, we believe there are opportunities for progress that do not compromise our values as public health professionals. We suggest three key steps to making progress.

A recent paper by Ralston and colleagues^1^ examined submissions to a World Health Organization (WHO) consultation on a tool to prevent and manage conflict of interest in nutrition policy. The authors documented widespread food industry opposition, not just to the details of the tool, but to the very idea of it. This response from the food industry should come as no surprise to the public health and policy worlds, as the WHO tool threatens to disrupt one of the food industry’s major sources of power – the ability not just to influence nutrition policy, but to determine it. Ironically, this opposition to a tool to manage conflicts of interest is a clear conflict of interest in itself. However, simply excluding food industry from the nutrition policy process is not necessarily the answer. To fully understand this ongoing battle for policy power, it is important to consider the multifaceted nature of the actors involved and the policy-making process itself.

Overall, food and nutrition policy-making is a complex and dynamic process that depends on many factors.^2^ It generally consists of three phases: a consultation phase, a policy decision-making phase and an implementation phase. Ideally, the consultation phase should be transparent and involve all relevant stakeholders, which may include food industry representatives. The policy decision-making phase should primarily involve government policy officers and decision-makers, with input from independent scientists (without conflicts of interest through associations with the food industry) where necessary. The implementation phase will again necessarily involve all relevant stakeholders, which may require co-operation from representatives of the food industry.

The most effective policy solutions to improving the nutritional status of populations are likely to be those that require little agency from (ie, make minimal demands on) individuals.^3^ These can take a number of forms although regulatory and legislative solutions, such as taxes on sugar-sweetened beverages, have been shown to be particularly effective in encouraging populations to consume healthier diets. However, these more effective policy tools are a direct threat to the commercial interests of certain food industry actors. When faced with this type of policy change, history shows that these food industry actors will use all possible resources to delay or subvert implementation.^4^

Whilst food industry involvement can be appropriate during the consultation and implementation phases of the policy process, there is an increasing trend for food industry actors to seek and secure involvement in the policy decision-making phase. Gaining a seat at the policy-making table or being given policy-making responsibility is one of the most effective and efficient uses of power by food companies as it means they can ensure that significant areas of policy will be developed and/or enacted in a manner that favours them.^5^

Examples of food companies being directly involved in policy decision-making can be seen in numerous countries around the world. Governments of the United Kingdom, United States, the European Union, and Australia have all attempted public-private partnership models of nutrition policy-making with the food industry. While these initiatives are often lauded by the governments responsible, evidence suggests they have little or no positive impact on public health.^6^

## Should We Exclude the Food Industry From Interacting With Governments?

 It is tempting to conclude that the food industry should be excluded entirely from interactions with governments. However, there are several problems with this position.

Firstly, the food industry is not homogenous. As Ralston et al suggest we “need to better differentiate between actors within the ‘food industry,’ an unhelpfully sweeping category that groups together such diverse entities as community-based farming cooperatives and multi-national companies, thus obstructing attempts to differentiate between those actors whose economic interests can and cannot be substantively reconciled or aligned with public health goals.” The tactics of influence designed to undermine public health are not coming from all actors within the food industry. Instead this behaviour is predominantly undertaken by trans-national corporations that largely manufacture processed and ultra-processed foods.^4,7^ These corporations have the deepest pockets with which to fund their policy influence and the most to lose from not doing so. They achieve powerful positions through numerous strategies including developing relationships with decision-makers, providing political donations, declaring themselves as experts within the sector, co-opting scientists and influencing the generation of scientific evidence.^5,8,9^

Secondly, as described above, it may be entirely appropriate to involve the food industry in the consultation and/or implementation phases of nutrition policy-making.^9^ For example, rolling out mandatory folate fortification in certain manufactured food products to prevent neural tube defects will require consultation with industry to determine which products can feasibly and most beneficially be fortified and to understand how long this process will take.^10^

## So What Should Be Done?

 While involving the food industry in certain aspects of the policy process without favouring their business goals may seem like an intractable problem, we believe there are opportunities for progress that do not compromise our values as public health professionals. There are three key steps to making progress.

Firstly, strategies can be adopted globally to protect nutrition policy-making processes from adverse interference from the food industry. The recent public consultation that occurred in Canada regarding the development of their *Healthy Eating Strategy *and the accompanying food guide serves as a model here. To ensure complete transparency, Health Canada instituted a policy which required any communication between Health Canada and stakeholders attempting to inform policy development to be published on the Health Canada website.^11^ Furthermore, industry-funded studies were excluded from the evidence base informing the strategy and members of the food industry were not allowed on the associated advisory body.^12^

 Secondly, researchers and civil society advocates need to work better together to hold food companies, as well as governments, accountable by examining existing policy-making processes and strategies of influence. Developing greater understanding of how we can more effectively counter the influence of harmful actors within the food industry will underpin this accountability process. This will require further research into effective advocacy strategies and innovative methods to regularly and comprehensively monitor the tactics of transnational food companies. It will also demand a better understanding of the multifaceted nature of actors within the food sector so that they can be more clearly differentiated in permitting their involvement in certain phases of the policy processes. We should not group all food sector actors together in the broad category of ‘food industry,’ more clearly assessing their interests in any particular policy proposal and excluding those whose interests clearly conflict with the policy goals.

Finally, for when it is appropriate to interact with relevant food industry actors during the consultation and implementation phases, public health and policy professionals need better guidance for how to do so in a manner that minimises the risk and consequences of conflicts of interest. Our research highlights that such guidance is desperately sought by scientists who need to interact with food industry actors in research^13,14^ and it makes sense that similar guidance is available for members states developing nutrition policy. The WHO tool to assess the risks associated with interacting with food companies^15^ – and work we have conducted to build on this for researchers – are tools that can help contribute to this.

 For too long this debate has been unhelpfully polarised, with some (often in public health sector) saying the food industry should be entirely excluded from the nutrition policy process, whilst others (often from private sector) supporting ever closer involvement. It is clear that there are occasions where nutrition policy-making can not only benefit from some interaction with food industry actors, but may be dependent on this. We propose that such interaction should be limited to the consultation and implementation phases of policy-making, and that greater guidance on managing conflict of interest could support policy-makers navigate this tricky terrain.

## Ethical issues

 Not applicable.

## Competing interests

 Authors declare that they have no competing interests.

## Authors’ contributions

 Conception and design: KC, JA, MW; Analysis and interpretation of data: KC, JA, MW; Drafting of the manuscript: KC; Critical revision of the manuscript for important intellectual content: JA, MW.

## Funding

 JA and MW are supported by the Centre for Diet and Activity Research (CEDAR), a UKCRC Public Health Research Centre of Excellence. Funding from the British Heart Foundation, Cancer Research UK, Economic and Social Research Council, Medical Research Council, the National Institute for Health Research, and the Wellcome Trust, under the auspices of the UK Clinical Research Collaboration, is gratefully acknowledged (grant number MR/K023187/1).

## Authors’ affiliations

^1^School of Public Health, University of Queensland, Herston, QLD, Australia. ^2^MRC Epidemiology Unit, University of Cambridge School of Clinical Medicine, Cambridge, UK.
